# Characterization and pathogenicity of Vero cell-attenuated porcine epidemic diarrhea virus CT strain

**DOI:** 10.1186/s12985-019-1232-7

**Published:** 2019-10-28

**Authors:** Yu Wu, Wei Li, Qingfeng Zhou, Qunhui Li, Zhichao Xu, Hanqin Shen, Feng Chen

**Affiliations:** 1 College of Animal Science, South China Agricultural University & Guangdong Provincial Key Lab of Agro-Animal Genomics and Molecular Breeding, Guangzhou, 510642 People’s Republic of China; 2 Wen’s Group Academy, Wen’s Foodstuffs Group Co., Ltd., Xinxing, 527400 Guangdong China

**Keywords:** Porcine epidemic diarrhea virus, Attenuation, Pathogenicity, Genome, Sequence analysis

## Abstract

**Background:**

Porcine epidemic diarrhea virus (PEDV) has caused enormous economic losses to the global pig industry. Currently available PEDV vaccine strains have limited protective effects against PEDV variant strains.

**Methods:**

In this study, the highly virulent epidemic virus strain CT was serially passaged in Vero cells for up to 120 generations (P120). Characterization of the different passages revealed that compared with P10 and P64, P120 had a higher viral titer and more obvious cytopathic effects, thereby demonstrating better cell adaptability.

**Results:**

Pathogenicity experiments using P120 in piglets revealed significant reductions in clinical symptoms, histopathological lesions, and intestinal PEDV antigen distribution; the piglet survival rate in the P120 group was 100%. Furthermore, whole-genome sequencing identified 13 amino acid changes in P120, which might be responsible for the attenuated virulence of P120.

**Conclusions:**

Thus, an attenuated strain was obtained via cell passaging and that this strain could be used in preparing attenuated vaccines.

## Background

Porcine epidemic diarrhea (PED) is an acute and highly infectious intestinal disease in pigs; it is caused by porcine epidemic diarrhea virus (PEDV), which has caused enormous economic losses to the global pig industry [1, 2]. PED is characterized by diarrhea, vomiting, dehydration, anorexia, weight loss, and high mortality in suckling pigs. Although PED also appears in summer, it mainly occurs in winter. PEDV infection causes different symptoms according to the pigs’ ages; however, in piglets, the symptoms of PEDV infection are particularly serious, including a high mortality rate [3].

PEDV belongs to the order *Nidovirales*, family *Coronaviridae*, and genus *Alphacoronavirus* and is an enveloped virus with a single-stranded, positive-sense RNA genome [2]. The whole PEDV genome is approximately 28 kb nucleotides (nts) long and has a 5′-cap and 3′-polyadenosyl tail; the genome also includes 5′- and 3′-untranslated regions and at least 7 open reading frames (ORF1a, ORF1b, and ORF2–6) [4, 5]. ORF1a and ORF1b encode the replicase polyproteins 1a and 1ab, respectively, which undergo autoproteolysis by viral proteases to form 16 nonstructural proteins (Nspl–16) [6], which participate in the basic mechanisms of viral RNA transcription and replication. ORF2–6 encode four structural proteins [fibrin (S), membrane protein (M), envelope protein (E), and nucleocapsid protein (N)] as well as coprotein ORF3 [7, 8]; these proteins are arranged in the genome in the following order: 5′-ORF (la/lb)-S-ORF3-E-M-N-3′ [9].

In 1978, the PEDV strain CV777 was identified as the cause of the PED outbreak in Belgium [10]. In October 2010, a highly pathogenic PEDV was discovered in China, which caused the worst outbreak on record and quickly swept across the country [11, 12]. The variant then caused a pandemic in the United States in spring 2013 and spread to Canada and Mexico. In addition, severe PED outbreaks occurred almost simultaneously in many Asian and European countries, such as Korea, Japan, Belgium, and France [13, 14].

Vaccination is considered effective in the prevention of PEDV infection on farms [15]. Several attenuated activated and inactivated vaccines for classical PEDV strains, such as CV777 [4], DR13 [16], and KPEDV-9 [17], have been developed and made commercially available in many countries [18]; however, the efficacy of these traditional vaccines against emerging PEDV strains is questionable because of the antigenic and genetic differences between the vaccine strains and the prevalent strains [13]. Therefore, there is an urgent need for a new PEDV vaccine against new variant strains.

In the present study, the CT strain was serially passaged in Vero cells. The growth kinetics and biological characteristics of the different passages were determined. In addition, 6-day-old piglets were used to assess the pathogenicity of these strains. Finally, the whole-genome sequences of the different passages were determined. A safe attenuated PEDV strain was developed in this study, thereby providing an important basis for the preparation of an attenuated vaccine.

## Methods

### Viruses, cells, and antibodies

The PEDV CT strain, which belongs to the G2b subgroup in China, was previously isolated by and stored at our laboratory [19]. Vero cells were obtained from the American Type Culture Collection (ATCC: CCL-81), regularly cultured in Dulbecco’s modified Eagle’s medium (DMEM) supplemented with 10% fetal bovine serum (Invitrogen, Australia) and 1% antibiotics (100 U/mL penicillin, 100 μg/mL streptomycin, and 25 μg/mL Fungizone®; Gibco™, USA), and maintained at 37 °C in a humidified 5% CO_2_ incubator. Mouse anti PEDV S monoclonal antibody and Y3-labeled goat anti-mouse IgG antibody were prepared and stored at our laboratory.

### Virus passages

Vero cells were grown in a T25 flask and washed thrice with phosphate-buffered saline (PBS) at 90% confluency. The cells were then incubated with 1 mL of the PEDV CT strain diluted 1:3000 in virus growth medium {DMEM supplemented with antibiotics (100 U/mL penicillin, 100 μg/mL streptomycin, and 7.5 μg/mL trypsin [Gibco])} for 1 h at 37 °C in a humidified 5% CO_2_ incubator. Then, 2 mL of the virus growth medium was added to the T25 flask, which was monitored daily for cytopathic effects (CPEs). When CPEs were observed in > 90% of the Vero cells, the flask was subjected to three cycles of freeze-thawing. The cells and supernatants were mixed by pipetting, aliquoted, and stored at − 80 °C. These harvested cells were then used as seed stock for the next passage. Finally, the cells were further passaged under each condition for up to 120 passages [5, 20].

### Immunofluorescence assay (IFA)

The Vero cells were inoculated with PEDV in 6-well plates at a multiplicity of infection (MOI) of 0.01. At 12 h post infection (hpi), the cells were washed thrice with PBS, fixed at room temperature (RT) with 4% paraformaldehyde for 15 min, and then permeabilized with 0.2% Triton X-100 (Solarbio, China) for 15 min. After washing as described previously, the cells were blocked with 1% bovine serum albumin (Solarbio) for 30 min at RT. The mouse anti PEDV S monoclonal antibody and Y3-labeled goat anti-mouse IgG antibody were then used as the primary and secondary antibodies, respectively. Finally, the cell nuclei were stained with 4′, 6-diamidino-2-phenylindole (Vectorlabs, USA) for 5 min at RT in the dark. The cells were then washed thrice with PBS and observed under a fluorescence microscope.

### Growth kinetics

Vero cell monolayers were separately inoculated with PEDV P10, P64, or P120 or were mock infected at an MOI of 0.01 in 6-well plates. The culture supernatants and cell lysates were collected at 6, 12, 18, 24, and 30 hpi. After one round of freeze-thawing, following the Reed–Muench method, the cell culture samples were analyzed in 96-well plates to determine the titration for 50% tissue culture infectious dose (TCID_50_/mL) [21].

### Genomic sequencing and analysis

Total RNA was extracted from the cell culture samples using an RNeasy kit (Magen, China). All primers used to amplify PEDV genomic fragments were designed and preserved at our laboratory [22]. Reverse transcription and polymerase chain reaction (PCR) were performed using the PrimeScript™ RT-PCR kit (Promega, USA) and PrimeSTAR® GXL DNA polymerase, respectively. The 5′- and 3′-end sequences were determined using a 5′- and 3′-competition kit (Takara). The PCR products were cloned into a pMD19-T vector using the TOPO® TA Cloning® Kit (Invitrogen, USA) and sent to Sangon Biotech (Shanghai, China) for sequencing. For each amplicon, more than three independent clones were sequenced to determine the accurate sequence of a specific genomic region. All sequencing results were then spliced and aligned using Lasergene SeqMan and Lasergene MegAlign, respectively. All reference sequences were obtained from GenBank and used for sequence alignment and phylogenetic analysis. All phylogenetic trees were constructed using MEGA7.0 software.

### Animal experiment design

Twenty-two 6-day-old crossbred (Duroc × Landrace × Big White) conventional piglets were obtained from Wen’s Foodstuffs Group Co., Ltd. (Guangdong, China). The piglets were determined to be free of antibodies against PEDV and confirmed to be negative for the major porcine enteric viruses PDCoV, PEDV, TGEV, and PRoV [23] via virus-specific RT-PCR of fecal swab samples [23, 24]. The piglets were then placed in separate cages and randomly divided into four experimental groups. Each group was placed in a separate room (Biosafety level 2). The piglets in each group were orally inoculated with 1 mL of the virus culture medium (1 × 10^6^ TCID^50^) or DMEM. The piglets were monitored daily for clinical signs of vomiting, diarrhea, and mortality throughout the experiment [25]. Fecal consistency was scored as follows: 0, solid; 1, paste; 2, semi-liquid; 3, liquid; and 4, death [26]. One piglet from each group was euthanized for histopathological examination at 3 days post infection (dpi). The remaining piglets were euthanized at the end of the study (10 dpi).

### Detection of PEDV shedding after challenge

Fresh fecal swabs were collected daily, diluted with PBS, stored at − 80 °C, and subjected to three freeze-thaw cycles. Viral RNA was extracted from the supernatant of the fecal swab samples as described previously. The amount of viral RNA in the fecal swab samples was determined using TaqMan real-time RT-qPCR with the following primers: sense, 5′-GAATTCCCAAGGGCGAAAAT-3′; antisense, 5′-TTTTCGACAAATTCCGCATCT-3′. A probe targeting the PEDV N gene (5′-FAM-CGTAGCAGGCTTGCTTCGGACCCA-BHQ-3′) was also employed. The thermal cycling parameters were as follows: 95 °C for 20 s followed by 40 cycles at 95 °C for 3 s and 60 °C for 30 s.

### Histopathology and immunohistochemistry (IHC)

The gross intestinal tract of each piglet was photographed at necropsy, and the intestinal tissue was collected and fixed with 4% paraformaldehyde. The fixed samples were sent to Guangzhou Saville for histopathology or IHC with PEDC S-specific monoclonal antibodies.

### Statistical analysis

In each experimental group, statistical significance was measured using one-way analysis of variance. Two-sided probability values < 0.05 (*P* < 0.05) were considered to indicate statistical significance. Correlations between the FC scores and fecal PEDV RNA drop titers were analyzed using Spearman’s rank correlation [26].

### Ethical approval

The animal study protocol was approved by the South China Agricultural University Committee of Animal Experiments (approval ID: SYXK-2014-0136). All experiments were performed in accordance with the recommendations of the Guide for the Care and Use of Laboratory Animals of the National Institutes of Health.

## Results

### Biological characteristics of PEDV P10, P64, and P120

**Fig. 1:**
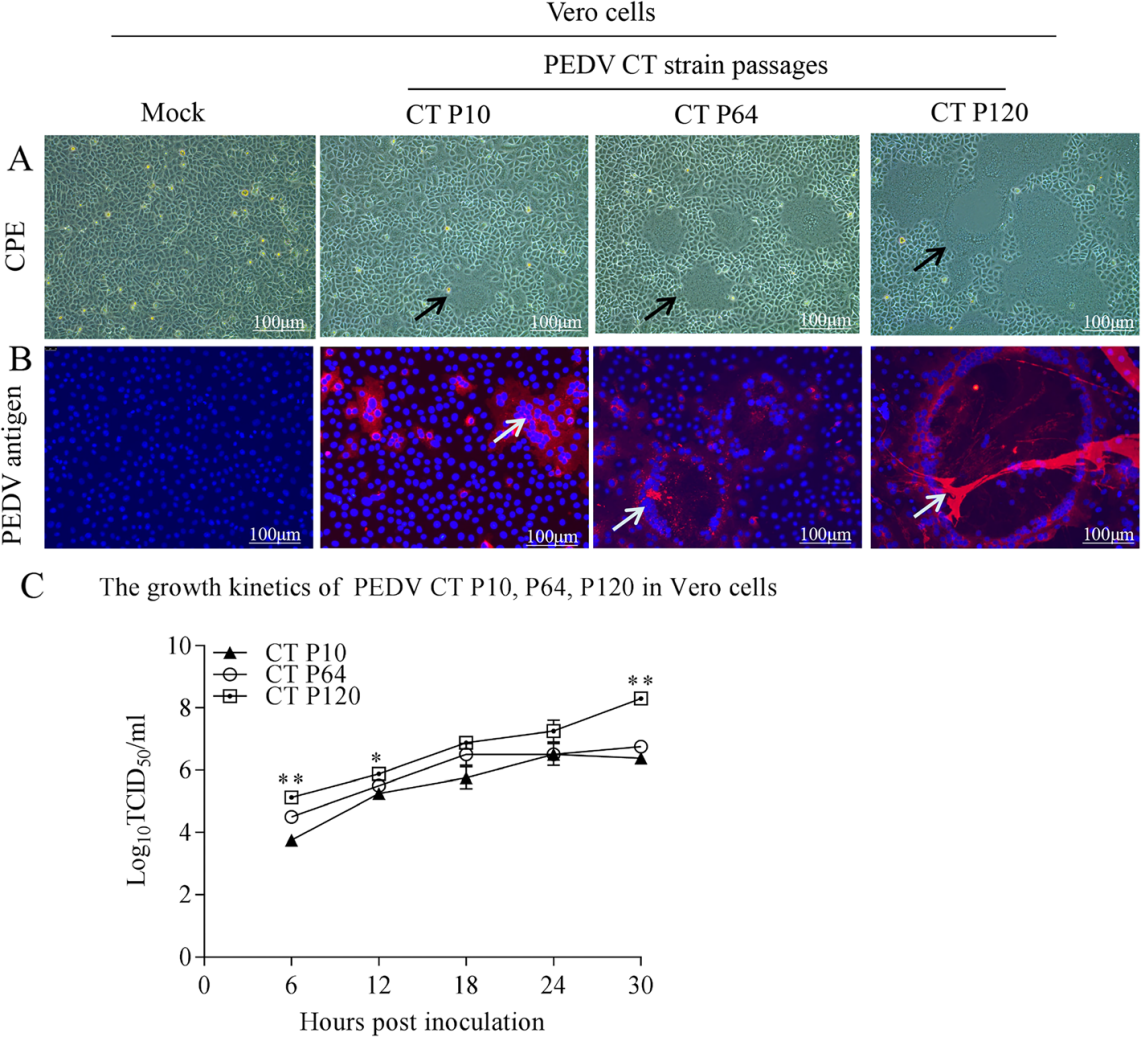
Biological characteristics of porcine epidemic diarrhea virus strains after 10, 64, or 120 passages. **a** Cytopathic effects of the three strains 12 hpi at an MOI of 0.01. **b** Immunofluorescence detection results for the P10, P64, and P120 strains in Vero cells infected at an MOI of 0.01 at 12 hpi. **c** Growth kinetics of the P10, P64, and P120 strains in Vero cells at an MOI of 0.01. Cell lysates were sampled at the designated time points and titrated using the titration for 50% tissue culture infectious dose infectivity assay. The asterisk means significant difference (***P < 0.001, **P < 0.01, and *P < 0.05). P10, PEDV after 10 passages; P64, PEDV after 64 passages; and P120, PEDV after 120 passages; MOI, multiplicity of infection; hpi, hours post infection

Our results indicated that syncytial, vacuole, and cell exfoliation were more obvious after infection with P120 than with P10 and P64 (Fig. 1a). The three different passage strains were confirmed by IFA using a specific monoclonal antibody against the PEDV S protein. Red signals were observed in the strain-infected Vero cells for all the three passage strains but not in the uninfected Vero cells at 12 hpi. Obviously, the cells infected with P120 produced more red PEDV antigens (Fig. 1b). In addition, growth kinetics indicated that P120 had a higher viral titer than the other two strains at all time points, indicating that P120 had better cell adaptability (Fig. 1c).

### P120 reduced clinical signs

**Fig. 2:**
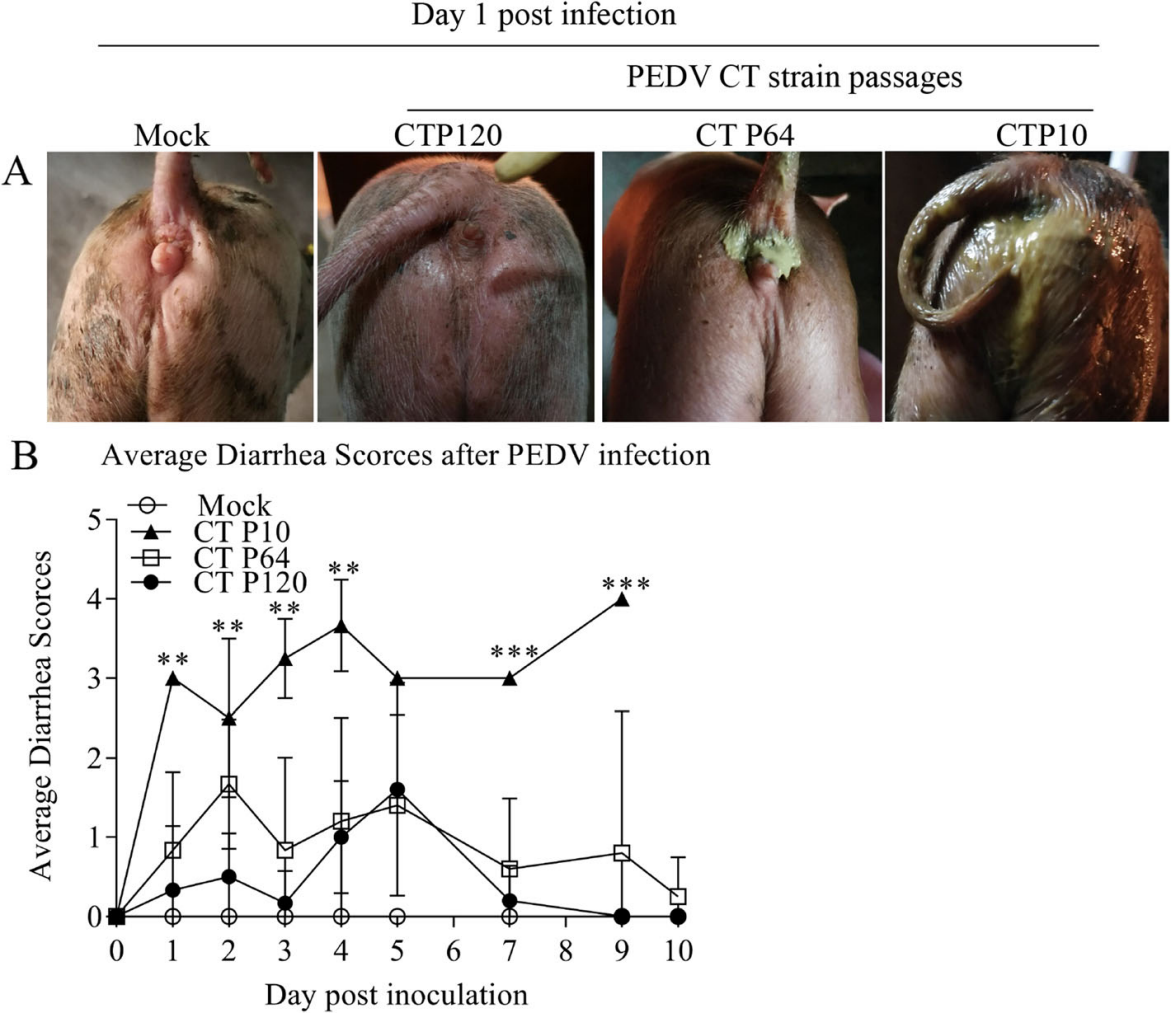
Clinical symptoms of piglets after PEDV infection. **a** Clinical symptoms of the four groups at 1 day post infection. **b** Average diarrhea scores of piglets after PEDV or mock infection during the entire experiment. ***P < 0.001, **P < 0.01, and *P < 0.05. PEDV, porcine epidemic diarrhea virus

Fecal swabs were collected from the piglets and scored for diarrhea during the experiment; the clinical signs of the piglets in each group were also recorded. At 1 dpi, severe diarrhea and vomiting were observed in the P10 group. In contrast, slight diarrhea and vomiting were observed in the P64 group. Interestingly, there were no obvious clinical symptoms in the P120 and mock groups (Fig. 2a). Regarding the lesion onset times in the groups, lesions appeared at 16, 24, and 48 hpi in the P10, P64, and P120 groups, respectively. Throughout the experiment, a high diarrhea score was maintained in the P10 group. In contrast, in the P64 and P120 groups, the diarrhea scores began to rise at 4 dpi, peaked at 5 dpi, and gradually returned to normal by 7 dpi (Fig. 2b).

### Differences in PEDV RNA shedding in the four experimental groups

**Fig. 3:**
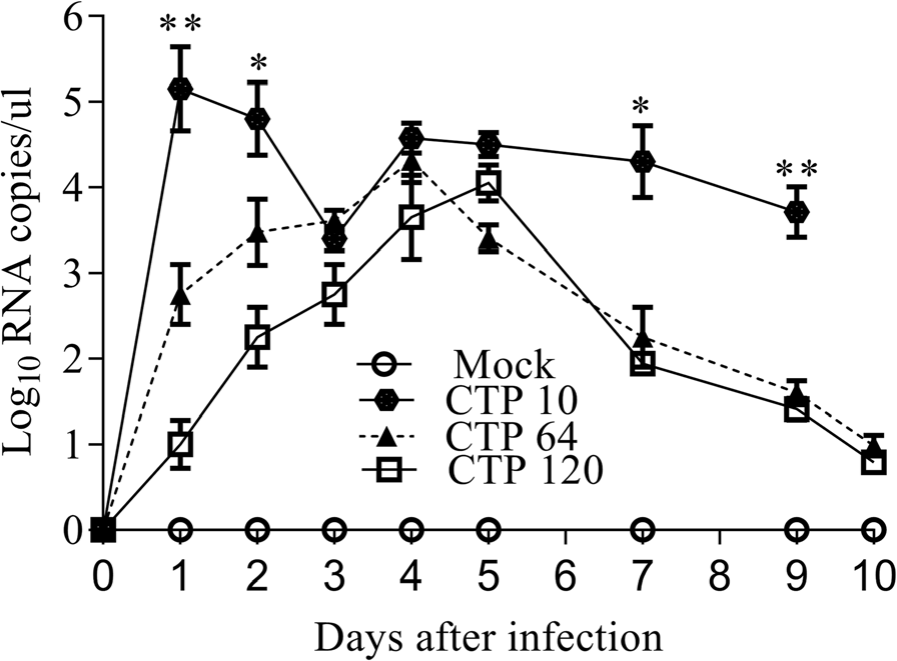
Viral RNA shedding of PEDV RNA shedding in piglet feces after different PEDV/ mock infection passages. ***P < 0.001, **P < 0.01, and *P < 0.05. PEDV, porcine epidemic diarrhea virus

PEDV RNA shedding was detected in fecal swab samples using RT-qPCR (Fig. 3). During the entire experiment, the P10 group maintained a high level of PEDV RNA shedding. In the P64 group, PEDV RNA shedding appeared at 1 dpi, peaked at 4 dpi, and gradually recovered by 7 dpi. PEDV RNA shedding was not observed in the P120 group until the second day after challenge; the viral shedding peaked at 5 dpi and gradually improved after 7 dpi. The entire experiment lasted for 10 days. The health of the piglets in the P120 and P64 groups had recovered by 10 dpi.

### PEDV CT strains reduced histopathological lesions at high passage levels

**Fig. 4:**
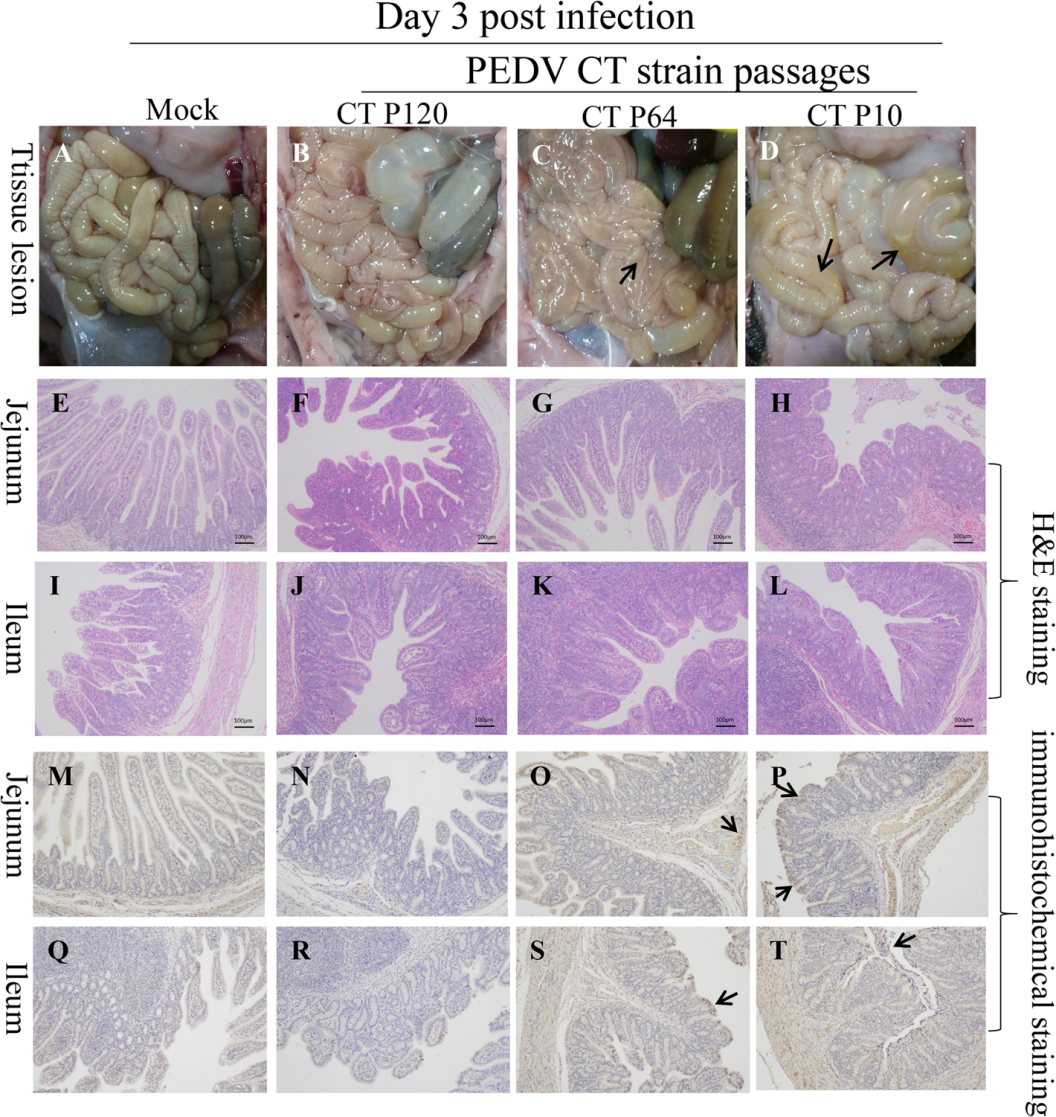
Histopathological and immunohistochemistry analyses. **a**–**d** Comparison of small intestinal lesions in different groups (Mock, P120, P64, and P10) at necropsy at 3 days post infection (dpi). **e**–**h** H&E-stained jejunum tissue sections of the different groups at 3 dpi. **i**–**l** **h**&**e**-stained ileum tissue sections of the different groups at 3 dpi. **m**–**p** Immunohistochemically stained jejunum tissue sections of the different groups at 3 dpi PEDV antigen is indicated by arrows). **q**–**t** Immunohistochemically stained ileum tissue sections of the different groups at 3 dpi (PEDV antigen is indicated by arrows). P10, PEDV after 10 passages; P64, PEDV after 64 passages; and P120, PEDV after 120 passages; **h**&**e**, hematoxylin and eosin; PEDV, porcine epidemic diarrhea virus

At 3 dpi, one piglet was selected from each group for dissection and sampling. At necropsy, intestinal tissue, particularly the small intestinal tissue, of the the P64 group piglet exhibited dilatation and transparency (Fig. 4a–d). In the P10 group piglet, the intestinal tissue lesions were more obvious and the intestine was filled with yellow fluid and mesenteric hyperemia (Fig. 4d). In contrast, the P120 group piglet exhibited only slight changes in the intestinal tract, and the control animal was extremely healthy with no pathological changes (Fig. 4b). The jejunum and ileum of each piglet were examined via histopathological and IHC analyses.

Histopathological analysis revealed severe atrophy and shedding of the intestinal villi of the P10 group piglet (Fig. 4h and l); in contrast, in the P64 group piglet, the intestinal villi were shortened and fused (Fig. 4f and j). Intestinal villus atrophy was mild in the P120 group piglet, and the examined control group piglet exhibited normal histopathology (Fig. 4e and i). In contrast, IHC revealed that the PEDV antigen was dominant in the cytoplasm of some segments of small intestinal villi. In the P10 and P64 group piglets, large amounts of the PEDV antigen were detected in severe lesions; in contrast, the antigen was hardly detected in the P120 group piglet (Fig. 4n and r); further, no PEDV antigen was found in the small intestine of the control group piglet (Fig. 4m and q).

### Survival of piglets after infection in the four experimental groups

**Fig. 5:**
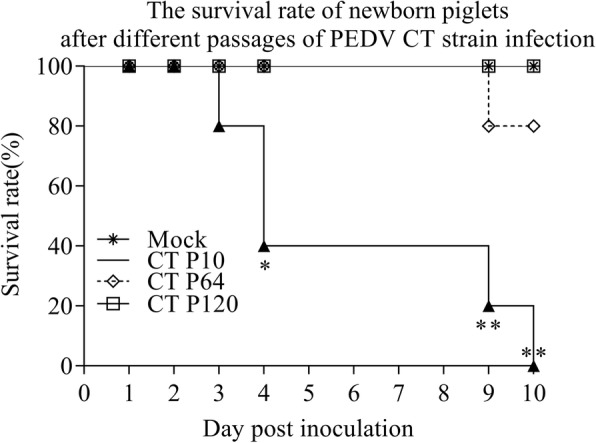
Survival rates of piglets infected with different passages of porcine epidemic diarrhea virus CT strain. **P < 0.01, and *P < 0.05

In the P10 group, one piglet died at 3 dpi and two others died at 4 dpi. In addition, one piglet each died at 9 and 10 dpi, respectively; thus, the survival rate in the P10 group was 0%. In the P64 group, the survival rate was 83.3% because only one piglet died at 9 dpi. No animals died in the P120 and control groups (100% survival rate for both groups) (Fig. 5).

### Sequence analysis of the different passage strains

**Fig. 6:**
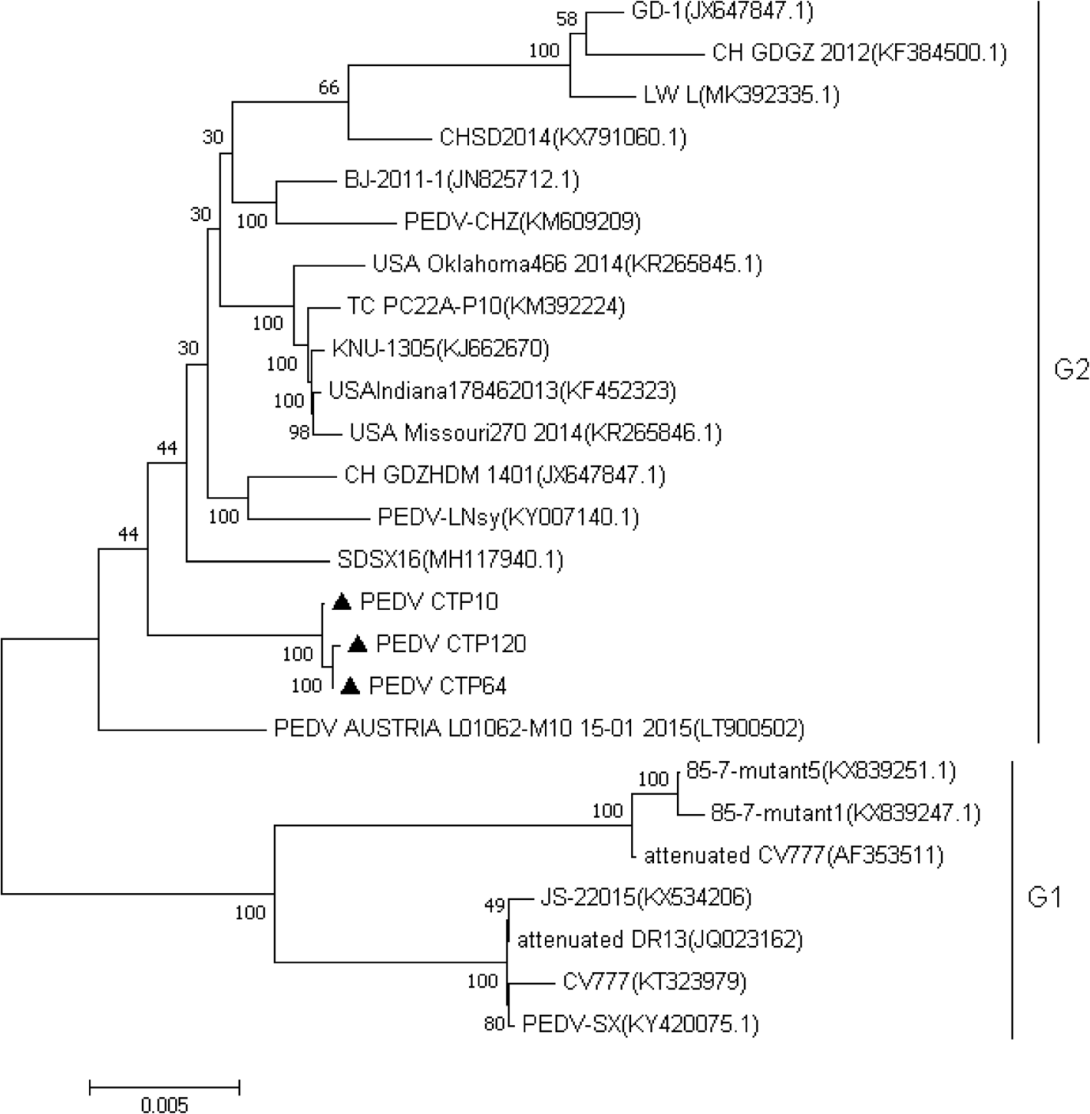
Phylogenetic tree analysis of the whole genomes of different PEDV strains. Phylogenetic analysis conducted for the whole-genome nucleotide sequence of the PEDV strains reported in our study and the reference PEDV strain. Using the MEGA7.0 software, the adjacency method was used to construct the tree. The numbers at the branch are the guided values (%) after 100 copies. The front ends of the three different sub-strains have been labeled. PEDV, porcine epidemic diarrhea virus

The genome length of the PEDV CT strain was determined to be 28,038 nts. Previous studies conducted at our laboratory have reported that the PEDV CT strain belongs to clade 6 of the G2b subgroup (Fig. 6). Table 1 lists the changes in the amino acids and nts of the different passage strains. P120 was observed to contain 14 base mutations, resulting in 13 amino acid mutations. The S gene is a determining feature of PEDV’s virulence and evolution [27]. Amino acid comparison indicated that there were only four amino acid mutations (D265A, F635R, S887R and C1362G) between P10 and P120. In addition, several nucleotide point mutations resulted in aa substitutions in ORF1ab (A1538S, T1945 N, D2813G, H2925Y, Y3302S and V4566I), ORF3 (T45 M), E (P70L) and M (G159D) proteins. We found that P64 had 10 base mutations (thus, 9 amino acid mutations). Moreover, compared with P64, P120 contained 4 base mutations (thus, 4 amino acid mutations) (Table 1). In addition, P120 had high homology with the CV777 vaccine strain (96.9%) and the attenuated DR13 strain (97.9%). The complete genome sequences of the CT strains described here have been deposited in GenBank under accession no.MN114121.

**Table 1:** Mutations of the nucleotide and amino acid in different passages of the PEDV CT strain

Gene	Nucleotide position	Amino acidposition	PEDV passages		
			CT P10	CT P64	CT P120
ORF1ab	4904	1538	GCC (A)	TCC (S)	TCC (S)
	6126	1945	ACT (T)	AAT (N)	AAT (N)
	8730	2813	GAT (D)	GAT (D)	GGT (G)
	9065	2925	CAT (H)	TAT (Y)	TAT (Y)
	10,197	3302	TAT(Y)	TCT (S)	TCT (S)
	13,987	4566	GTC (V)	GTC (V)	ATC (I)
S	794	265	GAT (D)	GCT (A)	GCT (A)
	1903–1904	635	TTT (F)	CGT (R)	CGT (R)
	2659	887	AGT (S)	AGT (S)	CGT (R)
	4084	1362	TGT (C)	GGT (G)	GGT (G)
ORF3	134	45	ACG (T)	ATG (M)	ATG (M)
E	209	70	CCC (P)	CTC (L)	CTC (L)
M	460	159	GGC (G)	GGC (G)	GAC (D)

## Discussion

PED outbreaks have caused huge economic losses to the pig industry in not only China but also the United States, Japan, South Korea, and other countries [28, 29]. Currently, vaccination is an effective solution to tackling the virus; however, owing to variations in the virus [18], the classical attenuated vaccine does not provide effective protection. Thus, the epidemic strains of the virus must be urgently studied to prepare a new attenuated vaccine.

In the classical method, attenuated vaccines are prepared via cell passaging [16, 17, 29, 30]. Some previous studies have reported that increased viral titers and enhanced cell adaptability are the characteristics of viruses with weakened virulence [4, 13, 26]. In the present study, we passaged the epidemic PEDV CT strain continuously in Vero cells for up to 120 generations and observed that P120 had better cell adaptability than the other two strains; thus, our findings illustrated that increased passaging increases the cell adaptability and titers.

Finding balance between cell and animal adaptability and then determining the best generation is the key to the preparation of attenuated vaccine candidate strains via continuous passaging [5, 25, 29]. Moreover, the adaptability of the strain to the host animal is characterized by the virulence of the strain, which is usually evaluated via pathogenicity testing of the animal infected with the virus. To prove that the strain virulence has been weakened, animal experiments were designed to confirm the strain [6, 8, 26]. Thus, the virulence of the three PEDV strains was assessed to confirm whether the P120 strain was weakened. According to data from animal experiments, the P120 strain could significantly reduce clinical symptoms, viral shedding, and histopathological changes in piglets. Therefore, our results illustrated that the P120 strain had relatively weak virulence and that compared with the P10 and P64 strains, it caused lesser damage to the piglets. Thus, it can be concluded that the P120 strain is safe and potentially useful as a candidate strain for the preparation of an attenuated vaccine.

To further assess the attenuation of the P120 strain, we sequenced the whole genomes of the three passage strains. And the method to prepare attenuated vaccine by cell passage can also get clues to the key virulence factors of the virus [29]. The PEDV S protein is involved in receptor binding, viral entry, neutralizing antibody production induction, and host cell fusion [9]. The S protein of PEDV has always been used as a marker of viral variation. Under the pressure of herd immunity, the S gene of PEDV mutates frequently, with some of the missense mutations altering viral antigenicity to aid in the virus’s escape from preexisting immunity. Thus, periodic vaccine updates may be required to ensure sufficient efficacy against emerging virus variants [31]. In this study, our result revealed that there is little variation in the S gene among these PEDV strains. Moreover, S protein is implicated in virulence, which was found out in the other studies that S protein can be variant readily while receiving immune pressure [32]. Mutations, including deletions and/or insertions, in the S protein may change the pathogenicity and tissue tropism of coronaviruses [33]. Comparing to the P10 strain, there was no nucleotides insertion and deletion in the S gene of the P120 strain. However, we found that there were four amino acid mutations (D265A, F635R, S887R and C1362G) between the P10 strain and the P120 strain. These mutations have not been reported previously. This finding indicates that these mutations in the S gene may be related to viral pathogenicity.

The ORF3 protein was reported to function as an ion channel and can prolongs S-phase, facilitates formation of vesicles and thus to regulate virus production [34]. It has been speculated that the deletion or mutation of a base in the ORF3 region promotes the adaptation of viral cells and changes in viral titers [35]. In this study, the T45 M mutation was detected in ORF3 of P120. Furthermore, mutations of amino acids in the ORF1ab, M, and E regions of P120 indicate that multiple mutation combinations in the genome cause complete decay of the PEDV strain via various molecular mechanisms [5]. These mutations found in ORF1ab, M, ORF3 and E proteins have not been reported previously. Further studies using reverse genetics are required to determine whether a particular or a combination of genetic changes (point mutations, deletions, and insertions) in PEDV strains alter viral infectivity, pathogenicity, and replication efficiency [26].

## Conclusions

In conclusion, an attenuated strain of PEDV with better cell adaptability and higher titers than the virulent strain was obtained via cell passaging. The results of our animal experiments illustrated that the pathogenicity of the P120 strain was reduced and that piglets infected with that strain had a 100% survival rate. Moreover, the results of whole-genome analysis revealed amino acid mutations in the P120 strain, which may contribute to the weakened virulence of the virus. Thus, the attenuated strain could be suitable for use in vaccine preparation.

## Data Availability

The datasets analyzed during this study are available from the corresponding author on reasonable request.

## Abbreviations

DMEM: Dulbecco’s modified Eagle’s medium
MOI: Multiplicity of infection
PBS: Phosphate-buffered saline
PCR: Polymerase chain reaction
PED: Porcine epidemic diarrhea
PEDV: Porcine epidemic diarrhea virus
RT: Room temperature
